# Guanylate-binding protein 1 inhibits nuclear delivery of pseudorabies virus by disrupting structure of actin filaments

**DOI:** 10.1186/s13567-023-01154-0

**Published:** 2023-03-14

**Authors:** Xiaohua Zhang, Qian Du, Guiyuan Chen, Yiyuan Jiang, Kai Huang, Linghao Li, Dewen Tong, Yong Huang

**Affiliations:** College of Veterinary Medicine, Northwest A&F University, Yangling, China

**Keywords:** Pseudorabies virus (PRV), GBP1, GTPase activity, serine/threonine protein kinase US3, actin filaments

## Abstract

The alphaherpesvirus pseudorabies virus (PRV) is the causative agent of pseudorabies, responsible for severe economic losses to the swine industry worldwide. The interferon-inducible GTPase guanylate-binding protein 1 (GBP1) exhibits antiviral immunity. Our findings show that there is a robust upregulation in the expression of porcine GBP1 during PRV infection. GBP1 knockout promotes PRV infection, while GBP1 overexpression restricts it. Importantly, we found that GBP1 impeded the normal structure of actin filaments in a GTPase-dependent manner, preventing PRV virions from reaching the nucleus. We also discovered that viral US3 protein bound GBP1 to interfere with its GTPase activity. Finally, the interaction between US3 and GBP1 requires US3 serine/threonine kinase activity sites and the GTPase domain (aa 1 to 308) of GBP1. Taken together, this study offers fresh perspectives on how PRV manipulates the host’s antiviral immune system.

## Introduction

Pseudorabies virus (PRV) is the aetiological agent of Aujeszky disease and has double-stranded DNA genomes that contain at least 70 open reading frames (ORF) [1–3]. Its genes are encoded in a cascade-like manner and divided into three major temporal classes: immediate-early (IE), early (E), and late (L) according to their dependency on viral DNA replication (L genes require the synthesis of viral DNA for their expressions). Viral US3 is a serine/threonine kinase protein present in the inner-tegument layer of the PRV virions [4]. It is a vital virulence factor of PRV [5] and functions in a wide range of processes during viral infection, including nuclear egress of viral capsids [6], promotion of retrograde transport [7], and enhancement of viral cell-to-cell spread [8, 9]. PRV US3 also impacts host cells in many aspects [10–13], including the phosphorylation and inactivation of the m6A writer complex [14], inhibition of histone deacetylase 1 and 2 (HDAC1/2) [15], rearrangements of the cytoskeleton [16], and disruption of various host defense mechanisms.

Viral infection activates the IFN signaling pathway resulting in the promotion of IFN expression and secretion and subsequently induces the expression of numerous IFN-stimulated genes (ISG), most of which defend cells from viral infection through various mechanisms by influencing the entire viral life cycle [17, 18]. Among the most abundant ISG, the IFN-inducible guanosine triphosphatase (GTPases) superfamily is conserved, including Guanylate-binding proteins (GBP), immunity-related GTPases, myxoma resistance proteins, and very large inducible GTPases [19]. GBP1 belongs to GBP and contains an N-terminal globular GTPase domain, a short middle domain, a C-terminal helical regulatory domain, and C-terminal CAAX motifs. The GTPase domain retains the main biochemical functions of GBP1 and its dimerization is necessary for sufficient GTP-hydrolyzing activity. The hydrolysis of GTP mediates the structural rearrangement of GBP1, which is important for the proper localization and formation of multimers. The helical regulatory domain allows for protein-protein as well as protein-lipid interactions. And the CAAX motifs are responsible for being isoprenylated to provide anchorage to endomembrane organelles [20]. In previous studies GBP1 was linked to antiviral activities against the dengue virus [21], influenza A virus (IAV) [22], hepatitis C virus (HCV) [23, 24], classical swine fever virus (CSFV) [25], Kaposi sarcoma-associated herpesvirus (KSHV) [26], vesicular stomatitis virus (VSV) [27], coxsackie virus, encephalomyocarditis virus (EMCV) [28], herpes simplex virus type 1 (HSV-1) [29] and porcine reproductive and respiratory syndrome virus (PRRSV) [30]. Whether GBP1 has antiviral activity against PRV, and its potential mechanism, remains unclear.

Herein, we discovered that both the transcriptional and protein levels of GBP1 were upregulated after PRV infection. Though GBP1 deletion exacerbated the severity of PRV infection, GBP1 overexpression considerably decreased it. The induced GBP1 prevents PRV virions from reaching the nucleus, which correlates with a disruption of the morphological integrity of the actin. Moreover, we found that the GTPase activity of GBP1 was related to restricting PRV infection. However, it was inhibited during PRV infection, which was concerned with viral US3. Overall, this research provides a deeper insight into PRV-induced innate immunity by characterizing the cross-talk between the virus and GBP1.

## Materials and methods

### Cells and viruses

PK-15 (porcine kidney 15 cell line) cells and HEK293T (human embryonic kidney 293 cells transfected with SV40 large T-antigen) cells were stocked in our lab [31]. These cells were propagated in Dulbecco minimum essential medium (DMEM) (12100-046; Invitrogen Carlsbad, CA, USA) supplemented with 10% heat-inactivated fetal bovine serum (FBS) (13 011 − 8611; Tianhang Biotechnology, Hangzhou, China) and plated in a fully humidified atmosphere containing 5% CO_2_ at 37 °C. PRV strain (Genbank: MH582511.1) was stocked in our laboratory.

### Reagents

4,6-diamidino-2-phenylindole (DAPI) staining solution (C1005) was purchased from Beyotime. Protein G-agarose (sc-2002) and protein A-agarose (sc-2001) were purchased from Santa Cruz. GTPase Kinetic ELIPA Assay Kit was purchased from Cytoskeleton (BK052).

### Quantitative PCR (Q-PCR)

RNA was extracted from cells using TRIzol reagent (solarbio), according to the manufacturer’s protocol. cDNA was synthesized from purified RNA using M-MLV reverse transcriptase (Invitrogen). The indicated mRNA levels were analyzed with Applied Biosystems QuantStudio 6&7 (Applied Biosystems, Grand Island, NY, USA) using SYBR-green (Takara). Specific primers about *GBP1*, *GAPDH*, *IE180*, *EP0*, *UL48*, *UL54*, *gB*, *gC*, and *gD* were used for qPCR: Sus scrofa *GBP1* forward primer, 5′-GGGGGATGTTGAGAAGGGTG-3′, reverse primer 5′- TCTCTCTGTCAGCTCGGTCA-3′, *GAPDH* forward primer, 5′-CTGGGCTACACTGAGCACC − 3′, reverse primer 5′-AAGTGGTCGTTGAGGGCAATG − 3′, PRV *IE180* forward primer, 5′-CATCGTGCTGGACACCATCGAG-3′, reverse primer 5′-ACGTAGACGTGGTAGTCCCCCA-3′, PRV *EP0* forward primer, 5′-GAGACCTGCCCATAAAGCCA-3′, reverse primer 5′-GGGAAGAGGATGAGCCGGTA-3′, PRV *UL48* forward primer, 5′-CACCCCCGCCTACCACCTC-3′, reverse primer 5′-CGG CTTTATACGCCCCACC-3′, PRV *UL54* forward primer, 5′-GTGGAACATGAGCGTCTCCC-3′, reverse primer 5′- GTGTTCAACGACGGCTTCC-3′, PRV *gB* forward primer, 5′-CCTCGTCCACGTCGTCCTC′, reverse primer 5′-GGCATCGCCAACTTCTTCC-3′, PRV *gC* forward primer, 5′-ACGTCTCGCTCGTCCTGTA′, reverse primer 5′- CGGGTAGTAGTCGCGGACG − 3′, PRV *gD* forward primer, 5′-CTGATCTCCGACCCGCAG′, reverse primer 5′-GCAGTCGGCGTACTCGATAA − 3′.

### CRISPR-Cas9-mediated GBP1 knockout in PK-15 cells

The CRISPR-Cas9 methodology was used to generate *GBP1* deletion cells in PK-15 cells as previously described [31]. Three pairs of gRNA specifically targeting the porcine *GBP1* were designed respectively using the optimized CRISPR design tool [32]. Oligonucleotide pairs of target sequences were phosphorylated and annealed. The acquired fragments were then ligated into the BsmB I sites of lentiCRISPRv2 plasmids (52,961; Addgene) and confirmed with sequencing analysis (Sangon Biotech). The correct recombinant plasmids with the packaging plasmids psPAX2 (12,260; Addgene) and pMD2.G (12 259; Addgene) co-transfected into HEK293T cells to obtain the lentivirus. After 72 h, the culture supernatant was collected to infect PK-15 cells for 48 h. Then, the cells were selected by puromycin (InvivoGen) at a concentration of 5 µg per mL. The *GBP1* KO cells were obtained after about 2 weeks. The cells obtained were subcloned into 96-well plates to get single-clone growth and saved as cell stocks.

### Immunoblotting and antibodies

Cells were lysed in a radioimmunoprecipitation assay (RIPA; solarbio) buffer containing 1mM phenylmethylsulfonyl fluoride and protease inhibitors (Sigma-Aldrich), and immunoblotting was performed as previously described [31]. The antibodies used in this study were as follows: anti-GAPDH (AB0036; Abways), anti-GBP1 (bs-13302R; Bioss), anti-HA (66006-2-Ig; Proteintech), anti-Flag (MA1-91878; Thermo Fisher), anti-Flag (701,629; Invitrogen), horseradish peroxidase (HRP)-conjugated anti-mouse IgG and anti-rabbit IgG (A16066 and 31 466; Invitrogen), anti-mouse IgG (H&L)-Alexa Fluor 488 (RS3208; Immunology), anti-mouse IgG (H&L)-Alexa Fluor 647 (RS3808; Immunology), anti-rabbit IgG (H&L)-Alexa Fluor 647 (RS3811; Immunology), anti-rabbit IgG (H&L)-Alexa Fluor 594 (RS3611; Immunology). Anti-gC and anti-EP0 of PRV polyclonal antibodies were produced in our lab.

### Immunofluorescence analysis

Cells were grown on glass coverslips and fixed with 4% paraformaldehyde. Next, it was permeabilized with 0.1% TritonX-100 for 15 min followed by blocking with 5% bovine serum albumin for 1 h. After blocking, the cells were incubated with the primary antibodies overnight at 4 °C, followed by incubation with the fluorescent secondary antibodies for 1 h at 37 °C. Cell nuclei were stained with DAPI. The actin filaments were stained with Cytopainter phalloidin-iFluor 488 for 1 h. The stained samples were photographed by a Leica TCS SP8 laser scanning confocal microscope.

### Immunoprecipitation assay

293T cells were seeded into 10-cm diameter dishes and transfected with the directed plasmids using the Lipofectamine 8000 reagent (Beyotime). Twenty-four hours after transfection, the cells were lysed with lysis buffer as described before [31]. Next, protein G/A agarose beads were added to the cell lysate supernatant for 1 h at 4 °C to pre-clean the nonspecific proteins. After pre-cleaning, the supernatant was incubated with the indicated antibodies overnight at 4 °C, followed by adding the protein G/A agarose beads for 2 h at room temperature. The beads were collected by centrifuging at 2000 × *g* for 10 s and washed three times with PBS. Finally, the proteins bound to the beads were eluted for Western blotting analysis with the appropriate antibodies.

### Animal experiments and Ethics statement

**Figure 1:**
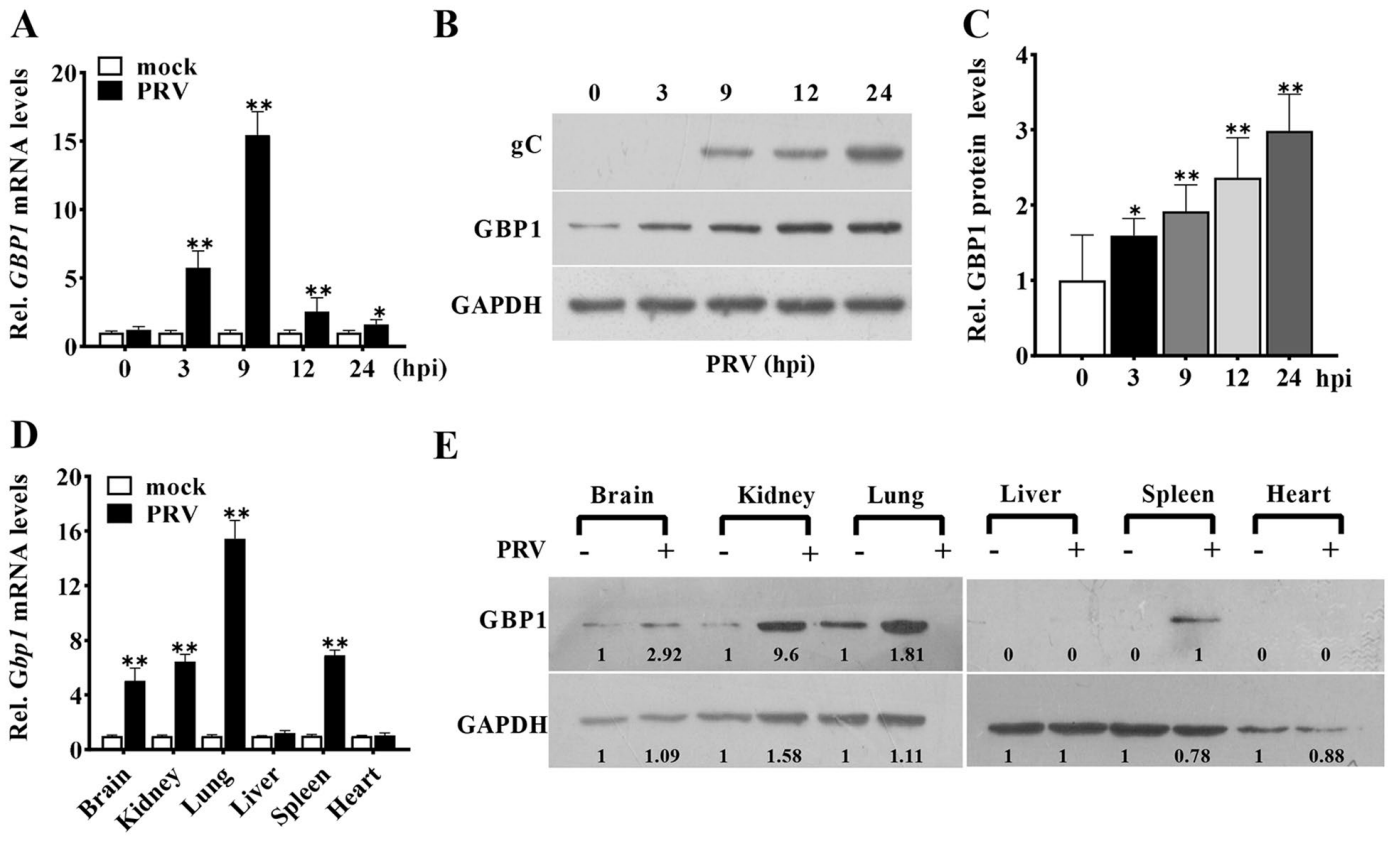
**The expression of GBP1 is elevated in PRV-infected cells and tissues.** Analyses of expression and translation of GBP1 in PRV-infected PK-15 cells (1 MOI) or tissues (50 µL of 5 × 10^5^ TCID_50_). **A** Relative change in amount of GBP1 mRNA measured by Q-PCR. Values versus mock-infected cells at the same points. **B** Representative Western blots of gC, GBP1, and GADPH. **C** Quantification of panel B. Values refer to 0 h cells. **D–E** Relative GBP1 mRNA levels **D** and representative Western blots of GBP1 and GAPDH **E** of different tissues with or without PRV infection. ** *P* < 0.01, compared with the same tissues in mock-infected mice.

Male C57BL/6 mice between 5 and 7 weeks old were purchased from the CHENGDU DOSSY EXPERIMENTAL ANIMALS CO., LTD. All mice were housed under the same conditions and treated similarly. For the experiment presented in Figure 1, mice were randomly divided into 2 groups (*n* = 6, per group), and inoculated with PRV (5 × 10^5^ TCID_50_) or mock (same volume of DMEM) for 72 h by intramuscular injection. The liver, lung, heart, kidney, spleen, and brain tissues were collected for further analysis.

The animal experiments with mice were approved by the Institutional Animal Care and Use Committee (IACUC) of Northwest A&F University (permit number: 20,210,616). The animal experimental operations were performed according to the Animal Ethics Procedures and Guidelines of the People’s Republic of China.

### Statistical analysis

GraphPad Prism 8 software was used to analyze all data. Data are expressed as the mean ± standard error of the mean (SEM) or the mean ± standard deviation (SD), and the results are representative of three independent experiments. Statistical comparisons between the two groups were analyzed using an unpaired Student *t-*test. One-way ANOVA was used to compare the differences among more than two groups, followed by the Bonferroni post hoc test or unpaired *t*-tests. The results were considered statistically significant at *P* values < 0.05 or < 0.01.

## Results

### The expression of GBP1 is elevated in PRV-infected cells and tissues

Since GBP1 is a major host cell factor involved in innate immunity, we characterized the kinetics of the mRNA and protein levels in PRV-infected PK-15 cells. When compared to the control, the *GBP1* mRNA levels were relatively upregulated from 3 to 24 h post-infection (hpi) (Figure 1A). Western blotting analyses proved that the GBP1 protein amount increased over time, similar to the change in RNA levels (Figures 1B, C). To further confirm this result, we identified the GBP1 expression levels in PRV-infected mice. As shown in Figure 1D, a remarkable increase of the *Gbp1* mRNA levels was found in PRV infected-tissues (brain, spleen, lung, and liver), which was absent in the liver and heart (Figure 1D). Similar to that, the GBP1 protein levels were elevated in the brain, kidney, spleen, and lung, but GBP1 was not detected in the liver and kidney (Figure 1E). Together, these data indicate that GBP1 expression is upregulated during PRV infection.

### GBP1 overexpression inhibits nuclear delivery of PRV virions, which correlated with a disruption of the morphological actin filaments

**Figure 2:**
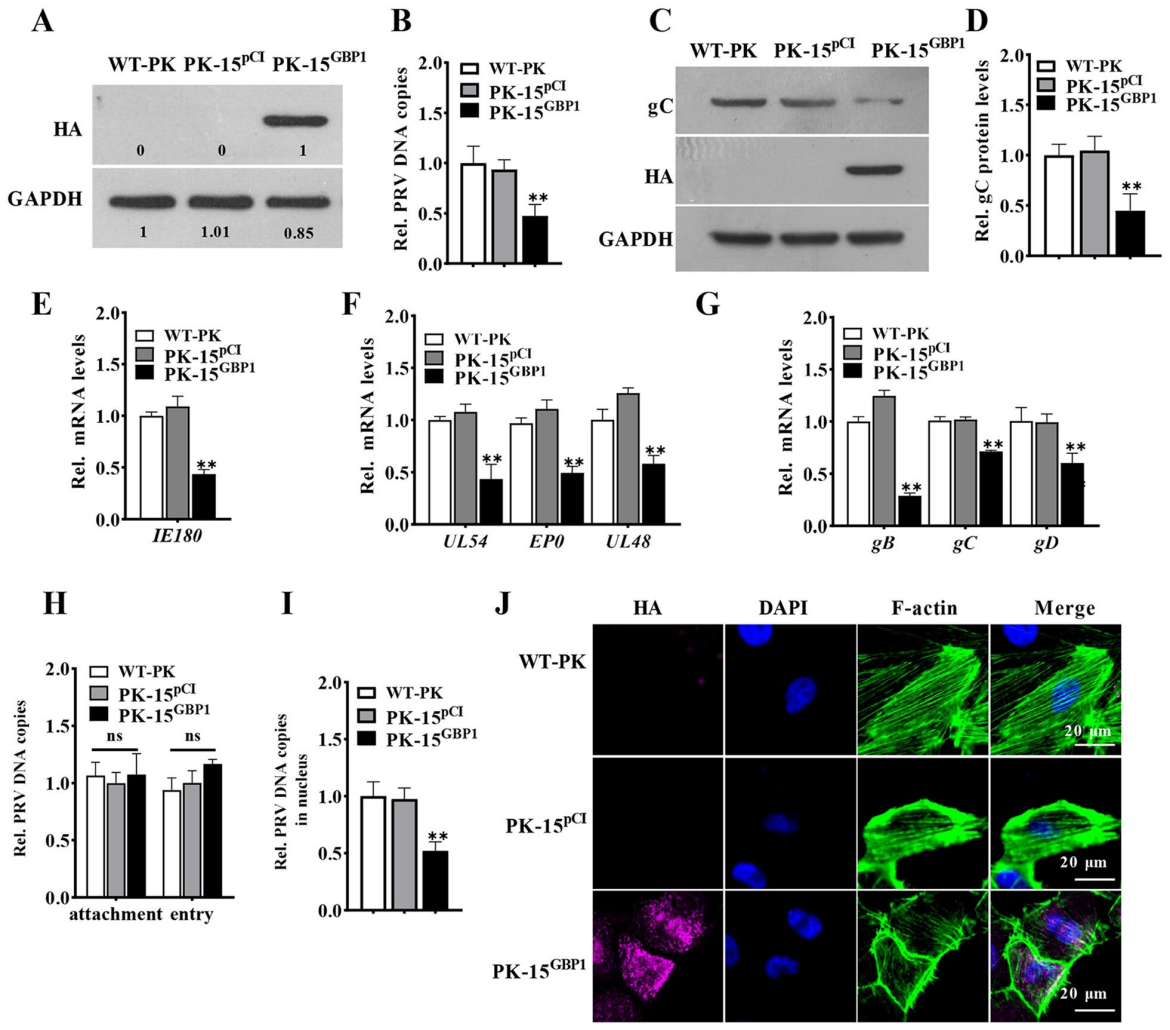
**GBP1 overexpression inhibits nuclear delivery of PRV virions, which correlates with a disruption of the morphological actin filaments.** PK-15 cells were stably transfected with pCI-GBP1 vector or the control vector pCI-neo, to establish PK-15^GBP1^ and PK-15^PCI^ cells selected by G418. **A** Representative Western blots of HA-GBP1 protein in described cells. **B, C** PK-15, PK-15^GBP1^, and PK-15^PCI^ cells were infected with PRV (1 MOI) for 24 h. Relative change in amount of the PRV genome copy number detected by q-PCR **B** Representative Western blots of HA-GBP1, gC and GAPDH. **D** Quantification of panel C. **E–G** PK-15 cells were harvested at the appointed times for detecting the mRNA levels of viral genes (immediate-early gene: *IE180* at 3 hpi, early genes: *EP0*, *UL54*, *UL48* at 4 hpi, and late genes: *gD*, *gB*, *gC* at 12 hpi). Relative change in the amount of viral gene mRNA. **H** These Cells were incubated with 10 MOI PRV for 1 h at 4 ℃, followed by three washes with cold PBS (for the adhesion group), and an additional hour of incubation at 37 °C (for the entry group). Next, total DNA was exacted and subjected to q-PCR. Relative change in amount of viral genome copy number. **I** Cells were incubated with 10 MOI PRV at 37 ℃ for 2 h, then the nuclear fraction was isolated to extract DNA for detecting the PRV genome copy number. Relative change in amount of viral genome copy number in the cell nucleus. **J** Representative immunofluorescent stain of DAPI (blue), HA-GBP1 (cherry), and F-actin (green). Scale bar, 20 μm. ** *P* < 0.01, versus the wild-type PK-15 cells (WT-PK).

To evaluate whether GBP1 is a restriction factor targeting PRV, we constructed a cell line stably expressing Flag-GBP1 (PK-15^GBP1^) and a PK-15 cell line transfected with blank vector (PK-15^pCI^) as the negative control. Western blotting results indicate that the GBP1 protein levels were significantly increased in PK-15^GBP1^ cells (Figure 2A). Upon infection, viral genome copies were decreased in the cells overexpressing GBP1, compared to that in the cells without GBP1 overexpression (Figure 2B). Similarly, the viral gC protein amount was also decreased in PK-15^GBP1^ cells after PRV infection (Figures 2 C and D). Additionally, we examined the changes in mRNA levels of viral immediate-early (IE) genes, early (E) genes, and late (L) genes by GBP1 overexpression. The mRNA levels of all representative genes were decreased in GBP1-overexpressing cells (Figures 2E–G), which suggests that GBP1 might function at the beginning of infection. However, the intracellular copies of the PRV genome had no significant difference across the group, indicating that GBP1 overexpression unaffected the adhesion and entry of PRV virions into cells (Figure 2H). We suspected that GBP1 might decrease the number of intracellular PRV copies at the post-entry stage. Thus, we checked whether the nuclear delivery of PRV virions was affected by GBP1. As shown in Figure 2H, GBP1 overexpression leads to a significant reduction of the viral genome copy number in the nucleus fraction (Figure 2I). The actin filaments can be destroyed by GBP1, whereas the intact cytoskeletal structure is essential for the nuclear delivery of PRV virions [22, 25, 26, 33]. Therefore, we tested the structure of actin filaments in WT-PK, PK-15^pCI^, and PK-15^GBP1^ cells. The results show that the normal structure of actin filaments was impaired in PK-15^GBP1^ cells compared to that in WT-PK cells and PK-15^pCI^ cells (Figure 2J). Collectively, these findings demonstrate that GBP1 inhibits the nuclear delivery of PRV virions, which relates to a disruption of the morphological actin filaments.

### Knockout of GBP1 enhances PRV infection in PK-15 cells

**Figure 3:**
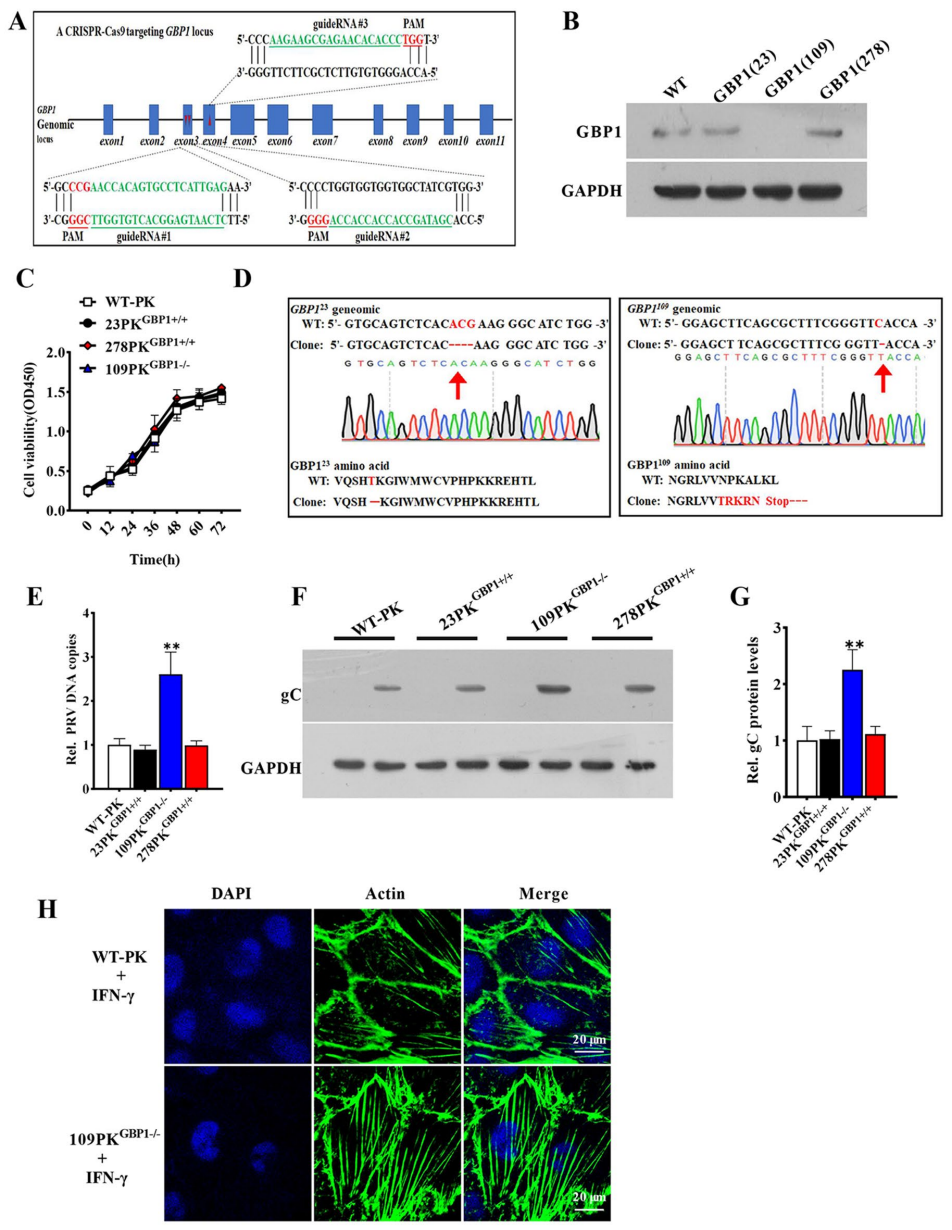
**Knockout of GBP1 enhances PRV infection in PK-15 cells. A** Schematic diagram representation of sgRNA targeting at the *GBP1* genomic region. Red arrows indicate the sgRNA-targeting sites (sites 1, 2, and 3) located on exon3, exon3 exon4. The protospacer adjacent motif (PAM) sequences are highlighted in red and the sgRNA targeting sites are in green. **B** Representative Western blots of GBP1. **C** The cell viability was measured by MTT. **D** Sequencing of GBP1 locus amplified from the 23PK-15^GBP1-/-^ and 109PK-15^GBP1-/-^ cells. Red arrows indicate deleted bases, red characters indicate deleted bases or mutated amino acids. **E, F** The described cells were infected with PRV (1 MOI) for 24 h. Relative change in amount of PRV copies **E** Representative Western blots of viral gC and GADPH **(F)**. **(G)** Quantification of panel F. ** *P* < 0.01, versus the wild-type PK-15 cells. **H** Representative immunofluorescent stain of DAPI (blue) and F-actin (green). Scale bar, 20 μm.

To further confirm the role of GBP1 during PRV infection, the GBP1 knockout PK-15 cells were constructed with the CRISPR/Cas9 genomic editing system. Three guide RNA (gRNA-23, gRNA-109, gRNA-278) were designed to target exon 3, exon 3, and exon 4 in the *GBP1* genome, respectively (Figure 3A). Western blotting analysis revealed that the GBP1 protein was absent in GBP1(109) cells but still present in the GBP1(23) and GBP1(278) cells (Figure 3B). Though genomic sequencing of the GBP1(23) cell clone (23PK-15^GBP1+/+^) showed a 3-nucleotide deletion leading to the deletion of threonine 75 site, the GBP1(109) cell clone (109PK-15^GBP1−/−^) displayed a single nucleotide deletion leading to the mutation of the 23–27 amino acid sequence and resulting in an early stop codon (Figure 3D). In contrast, genomic sequencing of GBP1(278) cell clone (278PK15^GBP1+/+^) was the same as the wild-type (WT) DNA sequence (data not shown). It should be pointed out that the knockout of GBP1 unaffected the viability of cells (Figure 3C). Then, the constructed three cell lines were used for determining the effects of GBP1 knockout on PRV infection. The viral genome copy number was higher in PRV-infected 109PK15^GBP1−/−^ cells than that in the wild-type PK-15 (WT), 23PK-15^GBP1+/+^ and 278PK15^GBP1+/+^cells (Figure 3E). Consistently, GBP1 knockout increased the amount of the viral gC protein (Figures 3F and G). GBP1 can be induced by IFN-γ [30], thus, we detected the actin morphology in PK-15 cells and 109PK^GBP1−/−^ cells after treatment of IFN-γ. IFA results proved that the normal structures of actin filaments were impaired with the 100 U/mL IFN-γ treatment in PK-15 cells, but this effect was largely abrogated in 109PK^GBP1−/−^ cells (Figure 3H). In conclusion, these data demonstrate that the deficiency of GBP1 promotes progeny virion production in PRV-infected cells, which might be associated with the normal structures of actin filaments.

### GBP1 inhibits the nuclear delivery of PRV virions requiring its GTPase activity

**Figure 4:**
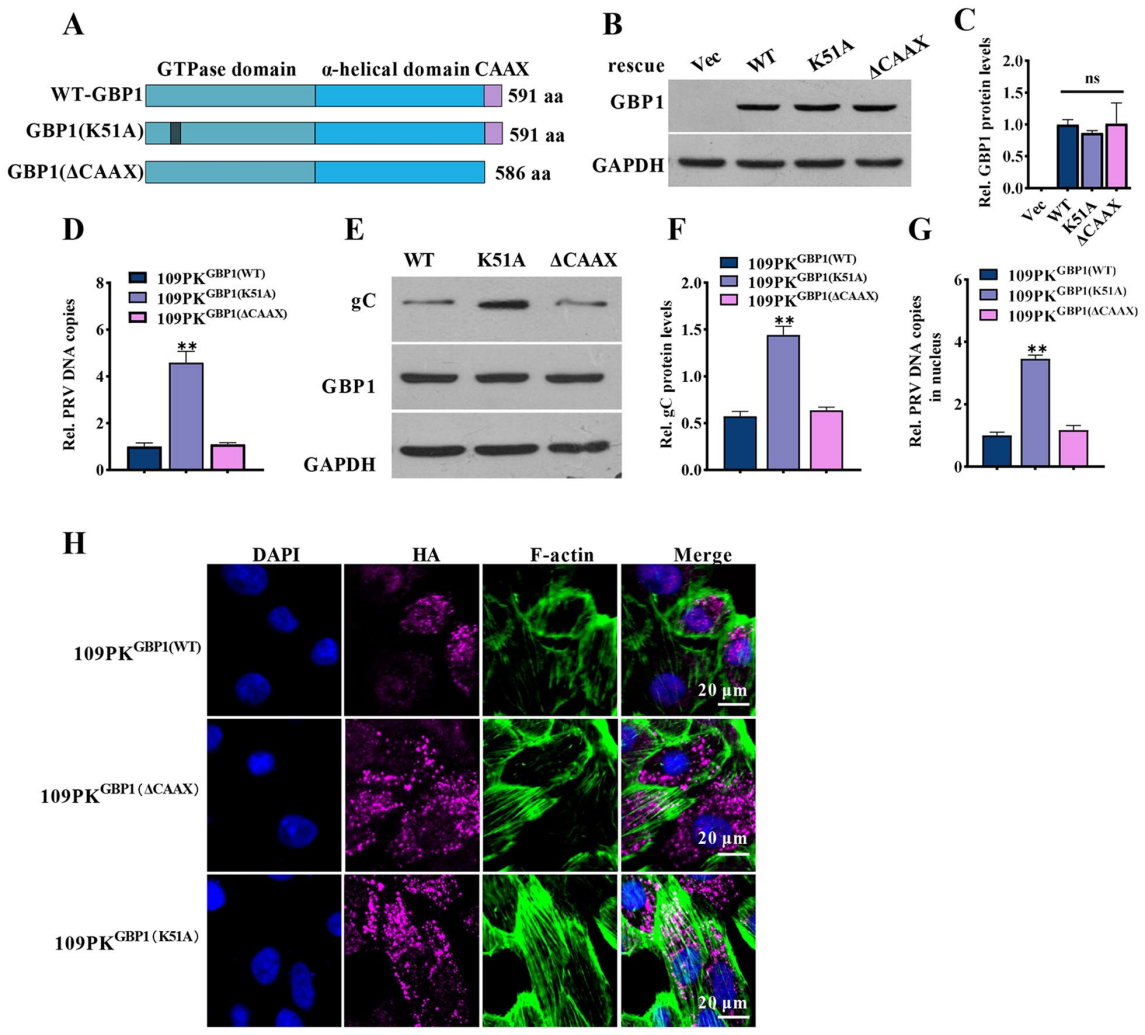
**GBP1 inhibits the nuclear delivery of PRV virions requiring its GTPase activity.** The wild-type GBP1(WT) and two GBP1 mutants GBP1(K51A) and GBP1(ΔCAAX) were constructed into the pCI-neo vector, subsequently stably transfected into 109PKGBP1^−/−^ cells, respectively defined 109PK^GBP1(WT)^, 109PK^GBP1(K51A)^ and 109PK^GBP1(ΔCAAX)^. **A** Schematic representation of full-length GBP1, two site-specific mutants GBP1. **(B)** Representative Western blots of GBP1 protein. **C** Quantification of panel B. **D–E** The three cell lines were infected with PRV (1 MOI) for 24 h. Relative PRV genome copies **D** and representative Western blots of GBP1, gC and GAPDH (**E**). **F** Quantification of panel E. **G** Described cells were incubated with 10 MOI PRV at 37 ℃ for 2 h. Relative PRV genome copy number in the cell nucleus. **H** Representative immunofluorescent stain of DAPI (blue), HA-GBP1 (cherry), and F-actin (green). Scale bar, 20 μm. ns, not significant; ** *P* < 0.01, versus 109PK^GBP1(WT)^ cells.

It has been reported that GBP1 contains a GTPase domain and a CAAX motif, both of which are significant for its antiviral activity [22, 25, 26, 33]. To identify the key domain concerned with restricting PRV infection, we generated three cell clones, 109PKGBP1^(WT)^ cells expressing wild-type GBP1, 109PKGBP1^(K51A)^ cells encoding GBP1 without GTPase activity, and 109PKGBP1^(ΔCAAX)^ cells expressing GBP1 that failed to locate to the Golgi (Figure 4A). The GBP1 expression was verified by Western blotting (Figures 4B and C). The number of viral genome copies in 109PKGBP1^(ΔCAAX)^ was comparable to that in 109PKGBP1^(WT)^ cells, while it was upregulated in 109PKGBP1^(K51A)^ cells (Figure 4D). Further to that, the gC protein levels were higher in the cells without GTPase activity (Figures 4E and F). In the nucleus of PK-15-GBP1^(K51A)^ cells, the viral genome copy number was also increased (Figure 4G). In addition, IFA results proved that the normal structures of actin filaments were impaired in 109PK^GBP1(WT)^ cells and 109PK^GBP1(ΔCAAX)^ cells, but not in 109PK^GBP1(K51A)^ cells (Figure 4H). In summary, these results suggest that GBP1 relied on its GTPase activity to suppress the nuclear delivery of PRV virions.

### The serine/threonine kinase activity of PRV US3 protein interfered with the GBP1 GTPase activity

**Figure 5:**
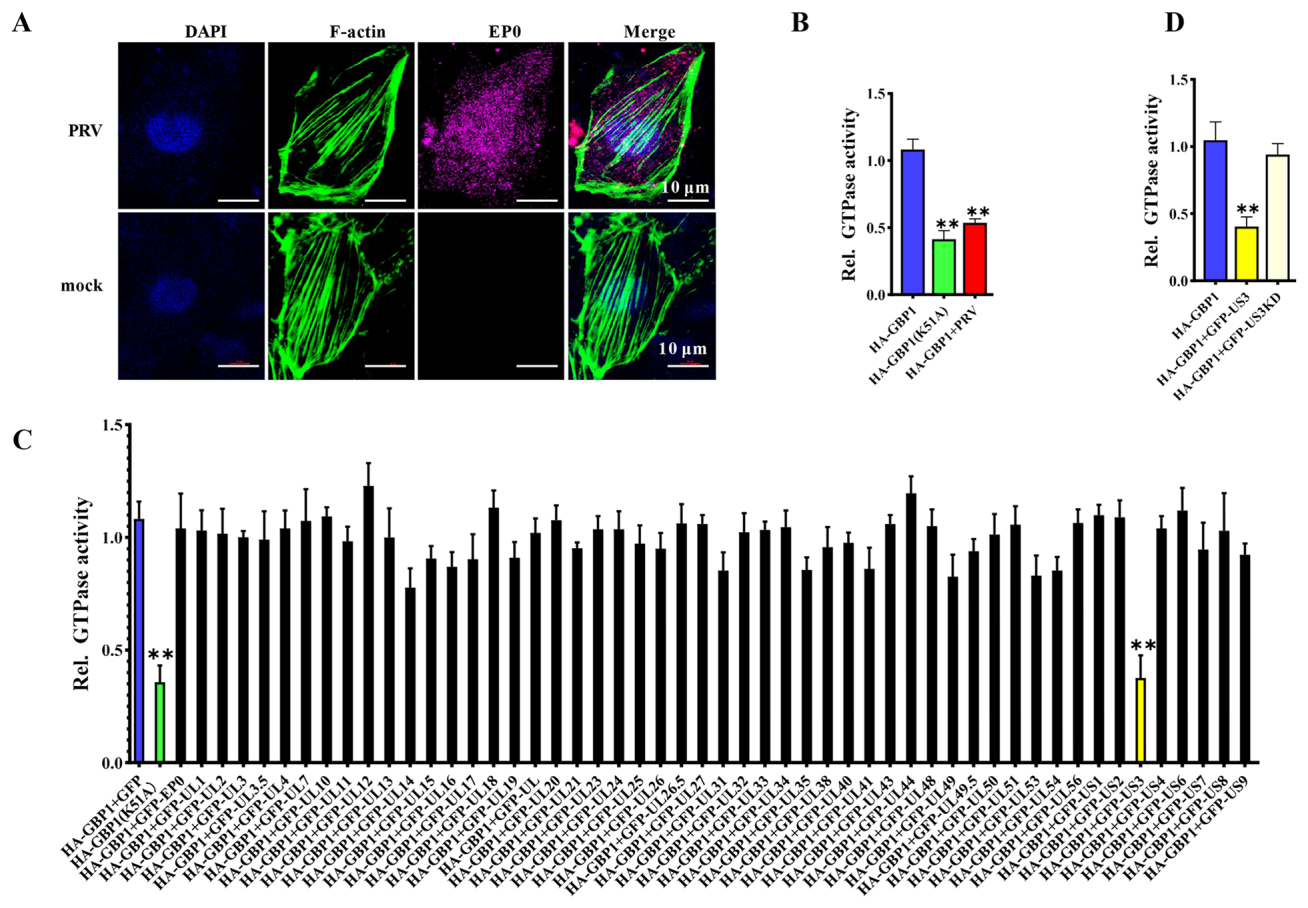
**The serine/threonine kinase activity of PRV US3 protein interfered with the GTPase activity of GBP1. A** The actin cytoskeleton was not damaged during the early stages of PRV infection (1 MOI, 4 h). Representative immunofluorescent stain of DAPI (blue), EP0 (cherry), and F-actin (green). Scale bar, 10 μm. **B–D** HEK293T cells were transfected with the indicated plasmids and GTPase activity was measured by enzyme-linked inorganic phosphate assay (ELIPA). The 293T cells transfected HA-GBP1(K51A) served as a control without GTPase activity. **B** The GTPase activity of GBP1 was suppressed during PRV infection. At 24 h post-transfection, cells were infected with or without PRV (1 MOI) for 2 h. Relative change in amount of GTPase activity.** *P* < 0.01, versus the cells expressing HA-GBP1. **C** The GTPase activity of GBP1 was suppressed by PRV US3. Relative change in the amount of GTPase activity. ** *P* < 0.01, versus the HA-GBP1 and pEGFP-N1 vector-transfected cells. **D** The US3 serine/threonine kinase activity was required for interfering with the GTPase activity of GBP1. Relative change in amount of GTPase activity. ** *P* < 0.01, versus the HA-GBP1 plasmid transfected cells.

We further explored whether PRV could counteract the antiviral activity of GBP1. At an early stage of PRV infection, actin filaments were still intact, similar to the uninfected PK-15 cells (Figure 5A), suggesting that the antiviral effect of GBP1 was antagonized during PRV infection. Since GBP1 expression was not decreased (Figure 1B), we speculated that its GTPase activity might be inhibited during PRV infection. ELISP assay shows that the GBP1 GTPase activity was decreased in PRV-infected cells, which proved the hypothesis (Figure 5B). Next, we constructed 52 plasmids encoding the indicated PRV protein to sift the protein that may inhibit the GTPase activity of GBP1. As seen in Figure 5, the GTPase activity was significantly reduced due to the expression of PRV US3. However, the expression of other viral plasmids or pEGFP-N1 had no significant effect on the GTPase activity (Figure 5C). PRV US3 is a multifunctional serine/threonine kinase protein, which can trigger the best characterized Rho GTPases RhoA, Rac1 and Cdc42 phosphorylation to regulate its activity [7, 17, 34]. To confirm whether US3 phosphorylates GBP1 to regulate its GTPase activity, we constructed GFP-US3KD plasmids encoding US3 without a functional kinase domain due to a K138A point mutation in the catalytic domain [35]. The results prove that the expression of GFP-US3KD did not affect the GTPase activity of GBP1 (Figure 5D). These results suggest that the serine/threonine kinase activity of viral US3 protein interferes with the GTPase activity of GBP1.

### The serine/threonine kinase activity sites of PRV US3 and the GTPase domain (aa 1 to 308) of GBP1 are necessary for the interaction between US3 and GBP1

**Figure 6:**
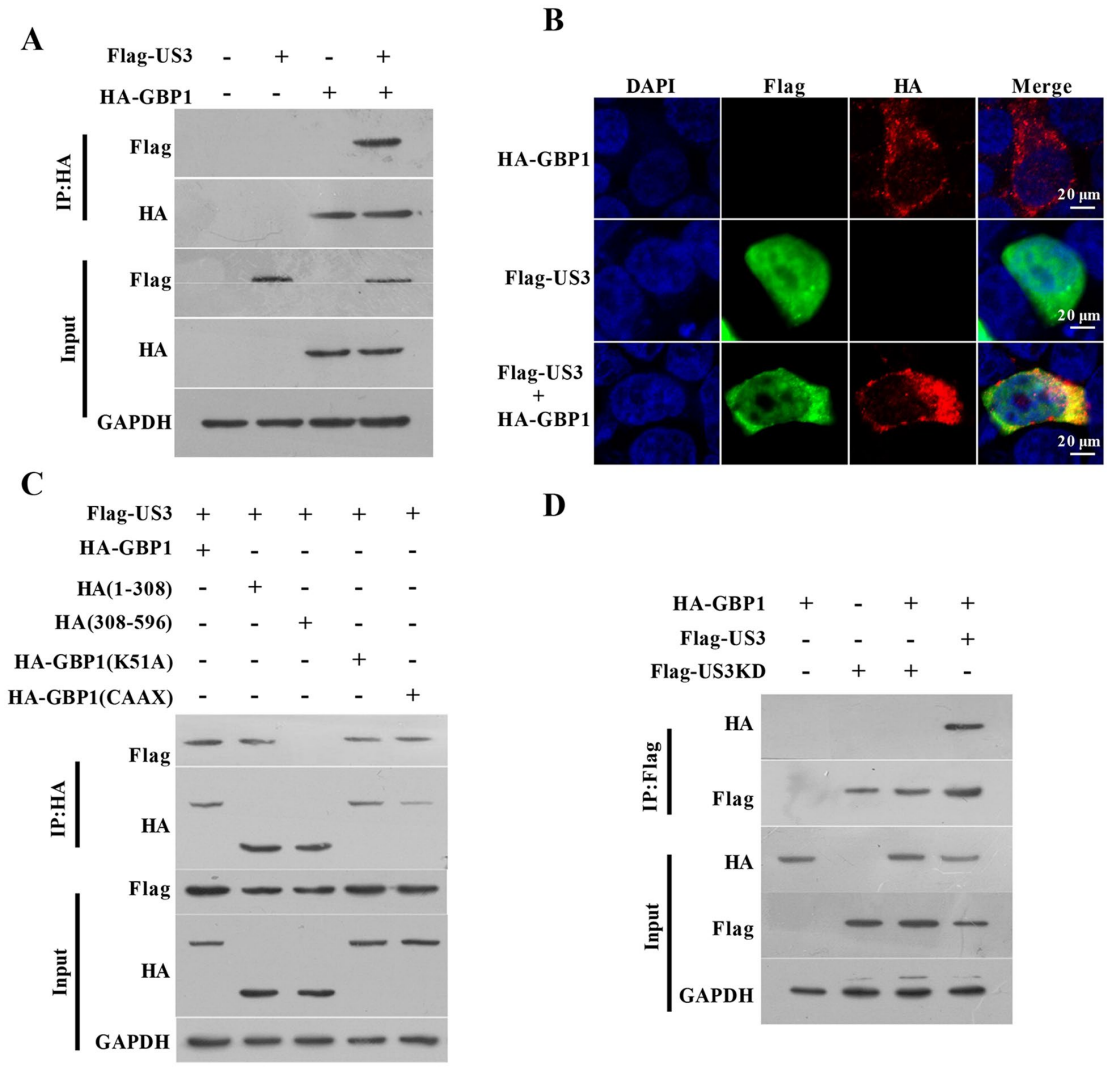
**The serine/threonine kinase activity sites of PRV US3 and the GTPase domain (aa 1 to 308) of GBP1 are necessary for the interaction between US3 and GBP1. A–B** PRV US3 interacts with GBP1 in transfected cells. HEK293T cells were transiently transfected with the indicated plasmids for 24 h. **A** Cell lysates were immunoprecipitated with an anti-HA antibody, followed by immunoblotting using anti-Flag, anti-HA, or anti-GAPDH antibodies. **B** Representative immunofluorescent stain of DAPI (blue), Flag (green), and HA (red). Scale bar = 20 μm. **C** GTPase domain (aa 1 to 308) interacted with US3. In the presence of Flag-US3 plasmids, HA-GBP1, HA-GBP1(1-308), HA-GBP1(308–591), HA-GBP1(K51A), or HA-GBP1(ΔCAAX) plasmids were transfected respectively into HEK293T cells. At 24 h post-transfection, cell lysates were immunoprecipitated with an anti-HA antibody. **D** The kinase activity sites of US3 are required for the interaction between US3 and GBP1. HEK293T cells were transfected with the described plasmids for 24 h, then were lysed for immunoprecipitation with Flag antibodies.

Since US3 can interact with several host cell proteins to induce their phosphorylation, we tested whether US3 was bound to GBP1. Both the co-immunoprecipitation assay (Figure 6A) and IFA assay (Figure 6B) show that HA-GBP1 could interact with Flag-US3, which confirmed our hypothesis. Four mutants of GBP1 were constructed to identify the regions of GBP1 required for US3 binding. As shown in Figure 6C, the co-immunoprecipitation assay showed that mutants HA-GBP1(1-308), HA-GBP1(ΔCAAX), and HA-GBP1(K51A) could interact with Flag-US3, whereas the HA-GBP1(308–591) was not able to interact with Flag-US3. These results indicate that the K51 site and CAAX domains were not required for interaction with US3, but the key domain for their interaction lies in the GBP1 N-terminus (GTPase domain). The US3 mutant plasmids (Flag-US3KD) were constructed to verify whether its kinase activity is required during the interaction. The results show that Flag-US3KD failed to interact with GBP1, indicating the importance of the kinase active sites of US3 for the interaction (Figure 6D). Thus, we conclude that the GTPase domain (aa 1 to 308) of GBP1 and the kinase activity sites of US3 are essential for the combination.

## Discussion

It has been demonstrated that GBP1 possesses antiviral functions, in turn, viral infection can affect its expression levels. For instance, GBP1 is produced in response to infections with IAV, CSFV, and HCV [22, 25, 26, 33, 36, 37]. Therefore, the first question we investigated was whether GBP1 expression was regulated during PRV infection. As seen in Figure 1, both the mRNA level and protein levels were upregulated in PRV-infected PK-15 cells and tissues, which told us that GBP1 might play a role in anti-PRV infection. Thus, the function of GBP1 in regulating PRV infection was further explored by comparing GBP1 wild-type, overexpressing, and knockout PK-15 cell lines. This research demonstrates that GBP1 overexpression inhibited the PRV infection, while the knockout of *GBP1* promoted it. Additionally, it was found that the mRNA levels of IE180 (PRV immediate-early gene) were decreased in GBP1-overexpressing cells (Figure 2E), suggesting GBP1 might function at an early phase of PRV infection. Though overexpression of GBP1 did not affect the adhesion and entry of PRV into cells (Figure 2H), a significant decline of virion number in the nucleus of infected cells was observed in GBP1 overexpression cells (Figure 2I), indicating that GBP1 might affect the intracellular transport upon PRV infection. It has been reported that GBP1 can disrupt the microfilament structure [38], which is essential for the entry of PRV virions into the nucleus [39]. This article shows that the overexpression of GBP1 indeed destroyed the microfilament structure (Figure 2J), which might prevent viral virions entering the nucleus.

Though the GTPase activity of GBP1 is required to combat a variety of viruses, the CAAX motifs, which are responsible for being isoprenylated to provide anchorage to endomembrane organelles also play some role in antiviral infection [22, 25, 26, 33]. Hence, the mutant GBP1 cell lines, 109PK^GBP1(K51A)^ and 109PK^(ΔCAAX)^ were constructed to detect whether the GTPase activity and CAAX motifs were required for inhibiting PRV infection. Our results show that GTPase activity, not the CAAX motifs, might be correlated with the inhibition of PRV infection (Figures 4D–G). In addition, without GTPase activity, GBP1 could not disrupt the microfilament structure (Figure 4H). All in all, GBP1 might disrupt the microfilament structure resulting in its disorder to inhibit the nuclear delivery of PRV virions, which relied on its GTPase activity.

Since the expression of GBP1 was increased (Figures 1A–E), actin filaments were not broken at the early stage of PRV infection (Figure 5A), we guessed that PRV might have a mechanism to counteract the GTPase activity of GBP1. Indeed, GBP1 GTPase activity was decreased after PRV infection (Figure 5B). We further constructed the indicated expression plasmids of PRV protein, sifting through the ELISP, and the GTPase activity was found to be inhibited by the serine/threonine kinase protein US3 (Figure 5C). Viral US3 is a multifunctional kinase protein, which can trigger the phosphorylation of various proteins to regulate their function. For example, RhoA, Rac1, and Cdc42, the best characterized Rho GTPases, can be phosphorylated by US3 to regulate many actin-driven processes [7, 16, 17, 34]. Our data show that without serine/threonine kinase activity, US3 could not exert anti-GTPase efficiency (Figure 5D). We guessed that US3 might also regulate the GTPase activity by phosphorylating GBP1. Viral proteins can bind to GBP1 to interfere with its GTPase activity and antiviral effect. For example, the NS5B of HCV interacts with the GTPase domain of GBP1, hence blocking its GTPase activity and antiviral effect to ensure the persistent infection and intracellular replication of HCV [23, 24]. Consistently, CSFV NS5A counteracts the antiviral activity of GBP1 by targeting its GTPase activity [25]. In the meanwhile, our results also identified that US3 was bound to the GBP1 (Figure 6A). Though the K51 of GBP1 is not the key binding site for the interaction of US3 and GBP1, the serine/threonine kinase activity sites of US3 and the GBP1 GTPase domain (aa 1 to 308) are necessary for the interaction between US3 and GBP1 (Figures 6B–D).

There are still many unanswered questions. For example, how the kinase activity of US3 regulates the GTPase activity of GBP1 has also not been explored. We have predicted that the GBP1 GTPase domain might contain sites that might be phosphorylated by US3, but this has not been studied in detail. Interestingly, the US3 protein has been shown to trigger RhoA phosphorylation to reorganize the actin cytoskeleton [16], which indicates that the role of US3 in the regulation of actin filament breakage is comprehensive and may be related to its location and expression levels. This research further broadens the antiviral spectrum and mechanism of GBP1.

Our project describes conclusively that GBP1 restricted the nuclear delivery of PRV virions by disrupting the actin filaments, which is dependent on its GTPase activity. During this process, viral serine/threonine protein kinase US3 bound to GBP1 to exert an antagonism effect. This study provides new insights into circumventing viral immune evasion strategies utilized by PRV.
